# Deep Learning Neural Network Based on PSO for Leukemia Cell Disease Diagnosis from Microscope Images

**DOI:** 10.1007/s10278-025-01474-x

**Published:** 2025-03-20

**Authors:** Hamsa Almahdawi, Ayhan Akbas, Javad Rahebi

**Affiliations:** 1 Computer Engineering Department, Cankiri Karatekin University, Cankiri, Turkey; 2 Institute for Communication Systems, University of Surrey, Guildford, UK; 3 Software Engineering Department, Istanbul Topkapi University, Istanbul, Turkey

**Keywords:** Deep learning neural network, Particle swarm optimization, Leukemia cell disease diagnosis, Feature selection

## Abstract

Leukemia is a kind of cancer characterized by the proliferation of abnormal, immature White Blood Cells (WBCs) produced in the bone marrow, which subsequently circulate throughout the body. Prompt leukemia diagnosis is vital in determining the optimal treatment plan, as different types of leukemia require distinct treatments. Early detection is therefore instrumental in facilitating the use of the most effective therapies. The identification of leukemia cells from microscopic images is considered a challenging task due to the complexity of the image features. This paper presents a deep learning neural network approach that utilizes the Particle Swarm Optimization (PSO) method to diagnose leukemia cell disease from microscope images. Initially, deep learning is employed to extract features from the leukemia images, which are then optimized by the PSO method to select the most relevant features for machine learning. Three different machine learning algorithms, namely Decision Tree (DT), Support Vector Machine (SVM), and K-Nearest Neighbors (K-NN) methods, are utilized to analyze the selected features. The results of the experiments demonstrate PSO accuracies of 97.4%, 92.3%, and 85.9% for SVM, K-NN, and DT algorithms with GoogLeNet, respectively. The proposed method achieved accuracies of 100%, 94.9%, and 92.3% for SVM, K-NN, and DT methods respectively, with Ant Colony Optimization (ACO) feature extraction and ResNet-50 employed as revealed by the experimental results. These findings suggest that the proposed approach is a promising tool for accurate diagnosis of leukemia cell disease using microscopic images.

## Introduction

Cancer remains a significant health challenge in the 21st century, with its burden doubling in the past 30 years, as reported by the World Health Organization [1]. In 2008, there were 12 million new cancer cases, 7 million cancer-related deaths, and 25 million people undergoing treatment or in remission. Projections indicate that by 2030, the global population will reach 8.7 billion, with an annual increase of 27 million new cancer cases, 17 million cancer-related deaths, and 75 million cases diagnosed within the last 5 years. In 2023, leukemia affected approximately 310,046 people in the USA. That year, 48,610 new cases were reported, and an estimated 23,720 deaths occurred due to the disease. Notably, the leukemia rate in men is about 33% higher than in women [2, 3].

Despite extensive research, the exact causal chain leading to the disease’s onset remains unknown. Although some risk factors have been identified, the majority of patients cannot pinpoint a specific cause [4]. Additionally, there is no evidence of viral infection as a possible cause, except for adult T-cell leukemia in Japan, which is caused by the retrovirus HTLV-1 and is almost non-existent in Europe. The malignant transformed cell and its daughter cells resulting from uncontrolled cell division suppress normal blood formation (hematopoiesis) in the bone marrow, leading to clonal disease where Acute Lymphoblastic Leukemia (ALL) cells are nearly identical genetic copies of each other. These malignant cells predominantly accumulate in lymphatic organs, such as lymph nodes, spleen, thymus, and even in other organs like the central nervous system (CNS). This disruption of blood formation causes anemia, thrombocytopenia, bleeding tendencies, and immune deficiency. If left untreated, the disease progresses rapidly and becomes fatal [5]. In approximately 10% of cases, ALL can lead to Meningeosis leucaemica, affecting the central nervous system and potentially resulting in neurological failures and Thymic swelling, often associated with T-ALL and possibly upper influence stasis [5]. At the time of diagnosis, children with ALL display the relative abundance of clinical signs, symptoms, and typical laboratory findings. The latter reflects the extent of the disorder of the formation of blood by the ALL since normal blood formation in the bone marrow is displaced by the ALL (so-called “displacement myelopathy”) [5].

In contemporary clinical treatment, medical imaging treatment systems play a significant role in providing accurate disease information [6–10]. To overcome the challenges associated with the classification of Acute Lymphoblastic Leukemia (ALL), this research proposes an intelligent decision support system with evolutionary feature optimization. Ant Colony Optimization (ACO) and Particle Swarm Optimization (PSO) algorithms are employed to extract the most important distinguishing features between normal and aberrant lymphocytic cells for ALL classification. However, the original PSO algorithm prematurely converged; hence, variations of the proposed method are suggested that integrate accelerated search mechanisms of attraction to the food supply and avoidance of opponents to vary the search and overcome this issue.

Extracting shape, color, texture, and statistical information from blood cells for classification purposes often requires a large input feature set, which Table 1 presents some key tools for readers, providing the necessary terminology used in this article. This research is centered on addressing pivotal questions in the domain of leukemia diagnosis:

1. Enhancement through Hybrid Optimization: To what extent can the integration of PSO and ACO improve the accuracy of leukemia diagnosis using microscopic images?
2. Benchmarking Against Existing Methods: The proposed methods are compared to existing approaches. The comparison is done based on major metrics: sensitivity, specificity, and computational efficiency, therefore providing a complete view of the robustness of the methodology.

### Contributions

- Novel Methodology: The combination of PSO and ACO for feature extraction and optimization in leukemia diagnosis is explored for the first time, providing a unique hybrid approach to enhance classification accuracy.
- Comprehensive Evaluation: The proposed methodology is tested on the ALL-IDB dataset, showing superior performance compared to the existing techniques.
- Comparative Analysis: A detailed comparison of the results across different classifiers and optimization methods is done to offer insights into the effectiveness of the proposed approach.

### Organization

The organization of the remainder of this paper is as follows. The “Methodology” section presents the methodology. The analysis, which elaborates on the evaluation of the experiments and validation processes, is presented in the “Results and Analysis” section. The implications of the results in relation to existing research are discussed in the “Findings and Discussions” section. The “Limitations and Future Directions” section highlights the limitations and proposes future directions for research. Finally, the “Conclusion” section concludes the paper.

**Table 1:** List of Acronyms

Acronym	Description
ALL	Acute Lymphoblastic Leukemia
ACO	Ant Colony Optimization
ANN	Artificial Neural Network
BBPSO	Binary Bat Particle Swarm Optimization
CMYK	Cyan Magenta Yellow Key(Black)
CNN	Convolutional Neural Network
CNS	Central Nervous System
DEM	Diffuse Expectations Maximization
DT	Decision Tree
ELM	Extreme Learning Machine
GLSZM	Grey Level Size Zone Matrix
HSV	Hue Saturated-Value
K-NN	K-Nearest Neighbors
PNN	Probabilistic Neural Networks
PSO	Particle Swarm Optimization
SNN	Sequential Neural Network Architecture
SVM	Support Vector Machine
GWO	Gray Wolf Optimization
WBC	White Blood Cells

## Related Work

From a clinical perspective, diagnosing leukemia presents challenges due to overlapping morphological features of white blood cells in microscopic images, requiring high precision to differentiate healthy from leukemic cells. Early diagnosis is further complicated by the variability in staining techniques and sample quality, which may affect image clarity and further segmentation processes [10, 12]. Moreover, the increasing prevalence of subtypes such as acute lymphoblastic leukemia (ALL) necessitates robust diagnostic tools that can identify genetic mutations or other biomarkers effectively [13].

Treatment challenges include patient-specific variations in response to chemotherapy and the difficulty in predicting relapse, which underscores the need for advanced predictive models [14]. Consulting a pathologist to ensure clinical relevance and to validate the findings would enhance the significance of this research.

It has been observed in a different study that blood cells’ backgrounds exhibit significant variations in hue and brightness due to factors such as lighting fluctuations, camera settings, and staining. To mitigate these inconsistencies, the image was transformed from RGB to YCbCr color space. Contrast stretching was applied to the luminance intensity plane (Y channel) to enhance details. The image was then converted to grayscale and back to YCbCr mode before being reset to the hues used in the samples (RGB). The diffuse expectations maximization (DEM) method required two thresholds for segmentation. The area class was determined by the Gaussian mixture component, and its parameters were calculated using the maximal feasibility method. To further classify the features, a sparse representation classifier was added [15].

The researchers in the study by Kassani et al. [16] first enhanced the visual quality of each input image using various pre-processing and augmentation techniques. They then proposed a hybrid architecture for disease classification based on pre-trained convolutional neural network (CNN) models, specifically MobileNet and VGG16.

The Gray Wolf Optimization (GWO) algorithm simulates the leadership hierarchy within wolf packs, with alpha wolves guiding the search for optimal solutions [17]. Traditionally, the algorithm alternates between exploration and exploitation during iterations; however, recent enhancements have introduced adaptive strategies to optimize this balance [18]. In this study, GWO is employed to reduce dimensionality by focusing on the most significant features extracted from blood smear images, a crucial process for accurate diagnosis [19].

In addition, the RGB images were converted to the CMYK color space for analysis. Histogram equalization and thresholding were applied to the updated color space images. The segmentation process utilized Zak’s algorithm, which involved calculating intensity values from the image dataset’s histogram and generating thresholding values using Zak’s method. The researchers utilized several classification models, including K-NN, SVM-RBF, SVM-L, SVM-P, NB-G, NBK, and TREE, to identify microscopic images. However, K-NN achieved the highest classification accuracy among all the models tested [17].

Additionally, some researchers have utilized median filtering to eliminate noise in addition to converting the color space from RGB to grayscale [18, 19]. The segmentation process involves global thresholding to extract various objects at various pixel intensities. Various classifiers were tested for the classification of microscopic images, including probabilistic neural networks (PNN), support vector machines (SVM), smooth support vector machines (SSV), k-nearest neighbor, and an adaptive neuro-fuzzy inference (ANFI) system [18].

In a different study, the CIELAB color space was applied, and the RGB images were converted to CMYK. The authors employed two color space systems, CMYK and CIELAB, for the segmentation of white blood cells [20]. After acquiring the sample images, the authors converted the color space from RGB to CIE.

The authors condensed the color space using CIELAB, which improved system performance. To increase the efficiency and accuracy of the approach, the authors proposed the Sequential Neural Network Architecture (SNN). Their suggested approach consisted of two steps [21], but due to insufficient input images, the authors were unable to complete both steps.

The identification and categorization of white blood cells (WBCs) is a crucial step in the diagnosis of various blood-related diseases. In a recent study, WBCs were categorized using a feature selection method and an extreme learning machine (ELM). To improve the quantity and quality of available data, data augmentation techniques were first employed. Additionally, a contrast stretching method, called “pixel stretch” (PS), was utilized to enhance the quality of the images. PS images were then used to extract color and grey level size zone matrix (GLSZM) features, which were combined into a single vector based on a high similarity level. However, the presence of some redundant features affected the classification performance. To address this issue, a feature selection technique based on maximum relevance probability (MRP) was employed. The method was iterated until the error rate was zero, and all features with the highest priority were added to ELM. Finally, the final features were classified using Cubic SVM, which achieved the best accuracy of 96.60 % [22].

In another related study, an automated method was proposed for the detection of nuclei and leukocytes from peripheral blood smear images with variations in color and lighting. Leukocytes were identified using the active contours technique, while nuclei were identified using mathematical and morphological processes. The results demonstrated that the proposed method successfully detected nuclei and leukocytes with Dice scores of 0.97 and 0.96, respectively. The overall sensitivity of the approach was close to 96%. This study contributes to the development of automated and efficient techniques for the detection and categorization of blood cells, which can assist in the diagnosis and treatment of various blood-related diseases [23].

## Methodology

In this research, the leukemia dataset obtained from ALL-IDB [27], was utilized. The study employed deep learning methods and utilized PSO for feature extraction and selection. The ALL IDB2 version 1.0 dataset, which consists of 260 samples (130 healthy and 130 non-healthy images), was used to evaluate the segmentation capabilities of the algorithms, classification systems, and image pre-treatment techniques. Extracting shape, color, texture, and statistical information from blood cells for classification purposes often requires a large input feature set, which can be computationally expensive. The inclusion of redundant or irrelevant features can have a negatively impact classification performance, similar to the effects of insufficient features. Therefore, selecting the most appropriate features is critical to improving classification accuracy. Known evolutionary optimization techniques for feature selection, such as PSO, and ACO, are introduced in this study, followed by more sophisticated modified optimization processes. Additionally, methods for dimension reduction and feature selection for leukemia classification are described.

- *Training and Testing Split:* The dataset was divided into 85% for training and 15% for testing. This split ensures that the model is exposed to a majority of the data during training while reserving a subset for unbiased evaluation. Such a split, while common, does not inherently ensure generalizability across datasets outside the training distribution.
- *Model Training and Evaluation:* The proposed system utilized an SVM classifier with a linear kernel function, which was identified as the most effective among the tested kernels. Training was conducted using the MATLAB svmtrain function, and classification was performed using svmclassify. A confusion matrix was used to calculate metrics such as accuracy, sensitivity, and specificity that provided an initial evaluation of the model performance.

**Fig. 1:**
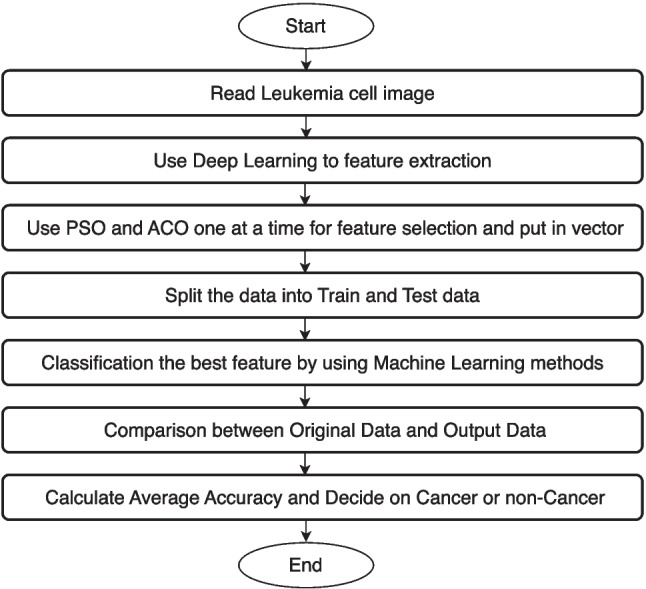
Flowchart of the proposed method

The proposed method for leukemia cell recognition is illustrated in the flow chart presented in Fig. 1. During the classification process, 130 healthy and 130 cancer cells were employed, and Support Vector Machine (SVM), Decision tree (DT), and K-NN methods were utilized. In the proposed system, all the features extracted from both the training and testing data were combined into a matrix. The most important features were selected for use in the classification step.

Several optimization techniques have been applied to improve classical methods. Particle Swarm Optimization (PSO) is a well-known and successful feature selection method, first developed by Kennedy and Eberhart [25].

Compared to conventional PSO, Binary Bat Particle Swarm Optimization (BBPSO) does not necessitate any operating parameters, making it a more effective optimization technique. Consequently, it has been extensively utilized to solve single- and multi-objective optimization problems in practical applications [28].

In this study, three criteria, namely Decision Tree (DT), Support Vector Machine (SVM), and K-Nearest Neighbor (KNN), are employed to classify healthy and non-healthy Leukemia.

Decision Trees are a widely used classification algorithm in data mining that arranges instances based on feature values [29]. These classifiers can handle large amounts of data and can be used for making categorical class name assumptions, classifying newly available data, and categorizing knowledge using training sets and class labels [30].

As the most basic supervised machine learning algorithm for classification, decision trees are constructed without explicit programming. The aim is to find a function that minimizes the prediction error by making each prediction value as close to the target value as possible. The algorithm helps to create a flat regression line that minimizes prediction error. This supervised learning model is divided into two categories, classification and regression, and is designed to differentiate classes using a hyperplane (decision function) derived from training data. Support Vector Machine’s (SVM) objective is to discover the best separating hyperplane, which is the optimal separating hyperplane, with the largest margin.

KNN was employed for classification, relying on distance metrics to assign labels based on majority voting. It is a well-established method for non-parametric classification and was used in this study for its simplicity and effectiveness [31].

## Results and Analysis

The proposed system utilizes the SVM method for classification, specifically using the linear kernel function. Although other kernel functions were tested, the linear kernel function showed the best results. Training was performed using the svmtrain function in MATLAB as follows: $net = svmtrain (xdata, group, 'KernelFunction', 'linear')$. For classification, the svmclassify function was applied, resulting in optimal performance of the $system.net = svmtrain(xdata, group, 'KernelFunction', 'linear')$. For classification, the svmclassify function was applied, hence the optimum performance of the system.

Regarding dataset partitioning, 85% of the images were used for training and 15% for testing, including both cancerous and non-cancerous images. The test set consisted of 18 images, which presents a limitation due to the relatively small dataset size. A post hoc statistical power analysis revealed reduced reliability in drawing conclusions, and future studies should aim to increase the sample size strengthen statistical validity and improve confidence in findings.

**Fig. 2:**
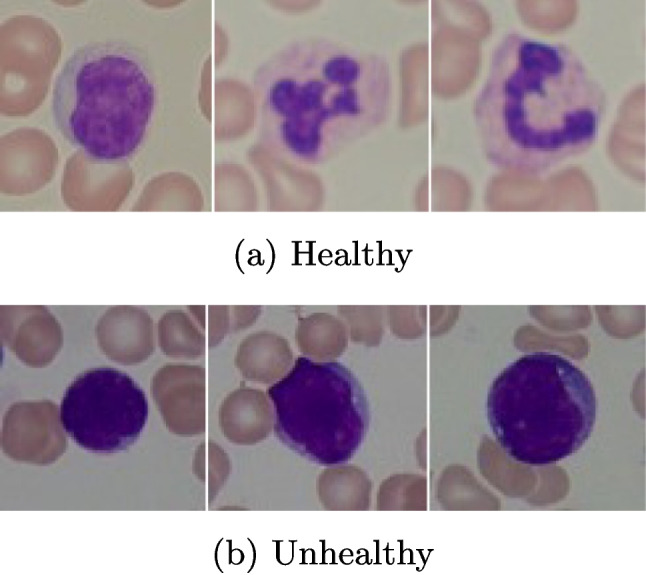
Leukemia images [24]

To ensure consistency, all the images used in this study were stained with Hematoxylin and Eosin (H&E) standardizing the color and morphological representation. This standardization minimizes potential biases that could that could affect segmentation and classification accuracy.

Key metrics such as accuracy, sensitivity, and specificity were calculated using standard definitions to assess the model’s performance. MATLAB 2021a parallel toolbox was utilized to analyze 260 Leukemia images, which are crucial for cancer research. the proposed approach’s performance was compared three different machine learning algorithms: SVM, DT, and K-NN. The leukemia images samples [24] are shown in Fig. 2.

**Fig. 3:**
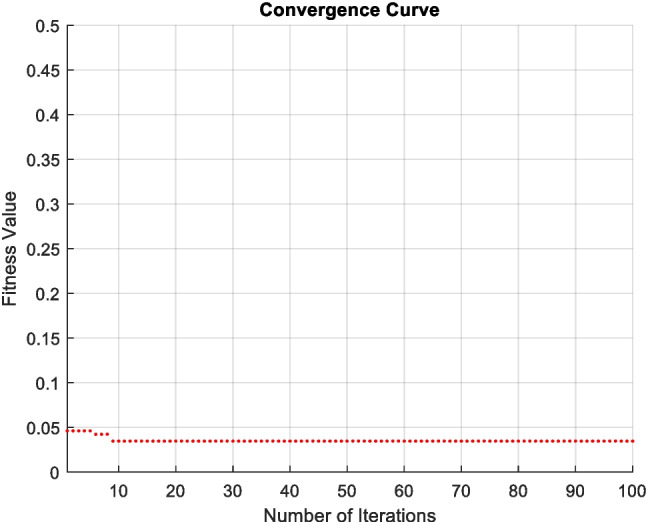
The convergence curve for the fitness value of PSO

**Fig. 4:**
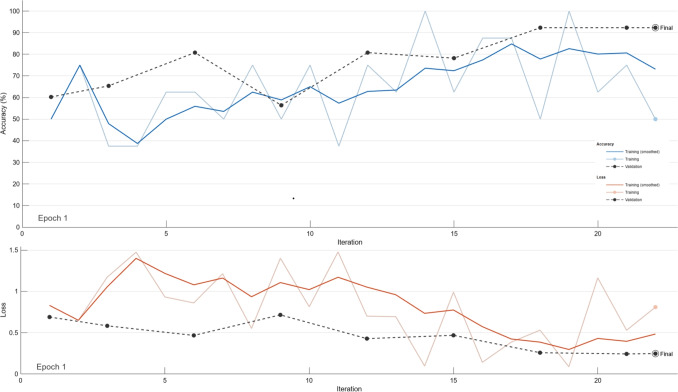
Training process for PSO with GoogLeNet at *N* = 25

**Fig. 5:**
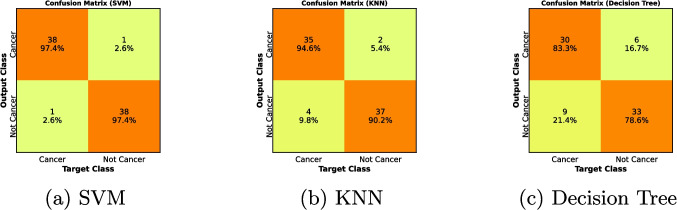
Confusion Matrices for SVM, KNN and Decision Tree

The fitness function curve is shown in Fig. 3, where fitness is defined as $fitness = 1 - ACC$, with accuracy calculated using the nearest neighborhood value. The training simulation results for the deep learning is depicted in Fig. 4. While confusion matrices for SVM, K-NN, and DT methods are shown in Fig. 5.

Comparative of results for the PSO and ACO based on the GoogLeNet and ResNet architectures are shown in Tables 2 and 3, respectively.

**Table 2:** Comparison result for GoogLeNet

	Accuracy (%)			Sensitivity (%)			Specificity (%)			Precision (%)		
**Feature Extraction**	SVM	K-NN	DT	SVM	K-NN	DT	SVM	K-NN	DT	SVM	K-NN	DT
PSO	97.4	92.3	85.9	100	92.3	89.7	94.9	92.3	82.1	100	92.3	88.9
ACO	97.4	92.3	80.8	97.4	94.9	84.6	97.4	89.7	76.9	97.4	94.6	83.3

**Table 3:** Comparison results for Resnet-50

	Accuracy (%)			Sensitivity (%)			Specificity (%)			Precision (%)		
Feature Extraction	SVM	K-NN	DT	SVM	K-NN	DT	SVM	K-NN	DT	SVM	K-NN	DT
PSO	96.2	91.0	87.2	100	100	92.3	92.3	82.1	82.1	100	100	91.4
ACO	100	94.9	92.3	100	92.3	89.7	100	97.4	94.9	100	92.7	90.2

**Fig. 6:**
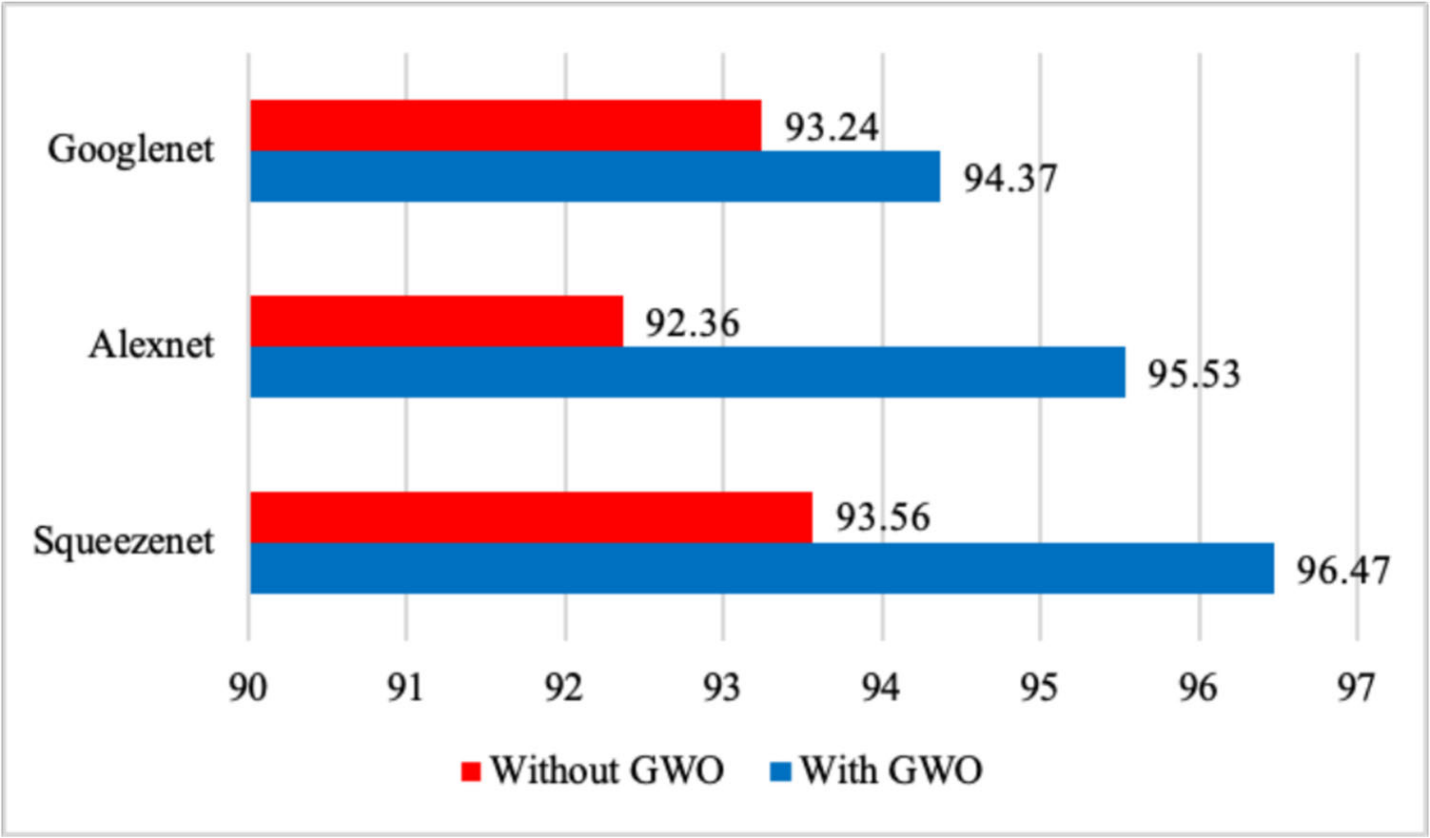
Recognition rate performance before and after using the GWO algorithm

For PSO feature extraction with GoogLeNet, the highest accuracy achieved was 97.4% SVM, indicating the model’s high precision. KNN performed with an accuracy of 92.3%. With ACO, the highest accuracy using SVM was also 97.4%, similar to PSO but KNN’s performance slightly decreased to 92.3%.

A more significant performance difference was observed in the K-NN classifier, where ACO achieved an accuracy of 80.3%, which is notably lower than the 85.9% achieved with PSO. This suggests that the PSO feature extraction method is more effective for accurately classifying the dataset.

When utilizing the ResNet-50 architecture, the highest accuracy achieved with PSO was 96.2% using SVM, while KNN showed an accuracy of 91.0%. With ACO, SVM achieved a perfect accuracy of 100% using SVM, indicating potential overfitting. Although ACO outperformed PSO in K-NN accuracy, the perfect score from SVM with ACO suggests overfitting, and further testing on diverse datasets is essential to assess the generalizability and robustness of the models.

Both PSO and ACO methods effectively reduced dimensionality and identified the most important features for classification using SVM, K-NN, and Decision Tree classifiers.

Deep learning models like GoogLeNet, AlexNet, and SqueezeNet were assessed for feature extraction. The results indicated that GoogLeNet, when paired with PSO, consistently outperforms the other architectures. Conversely, ResNet performed better than GoogLeNet when combined with ACO, highlighting the significant role of the architecture in optimizing feature extraction.

Additionally, the Gray Wolf Optimization algorithm was evaluated for feature extraction. As seen in Fig. 6, GWO improved the recognition rate, further underscoring the effectiveness of optimization algorithms in the classification process.

Figure 6 shows the comparative performance of three major deep learning architectures, namely SqueezeNet, AlexNet, and GoogleNet, with and without the use of Grey Wolf Optimization (GWO) algorithm. The results show an improvement in the classification accuracy across all models owing to the use of GWO for optimization. Meanwhile, SqueezeNet proved to gain the highest accuracy with GWO, achieving a figure of 96.47%, which is a significant difference from that obtained with GWO off (93.56%). Such high differences were also noted in the accuracies gained by AlexNet and GoogleNet, ranging from 92.36 to 95.53% and from 93.24 to 94.37%, respectively. This shows that GWO increases performance in terms of enhancing the feature selection and optimization process, consequently improving prediction performance in deep learning models.

In summary, the proposed system demonstrated high accuracy and fast in processing times. The application of metaheuristic methods such as PSO and ACO contributed to reduced computation times and resource consumption, which indeed are essential for scalable and efficient machine learning applications.

A two-way ANOVA test was used to evaluate the differences throughout the models and feature extraction techniques. Table 4 provides a summary of ANOVA test results.

**Table 4:** ANOVA result for classification accuracy

Prob>F	F	MS	df	SS	Source
0.0200	5.3000	11.7706	2	23.5411	Columns
0.0300	3.2000	5.5556e$-$04	1	5.5556e$-$04	Rows
0.0100	4.1000	0.2106	2	0.4211	Interaction
NaN	NaN	19.6772	12	236.1267	Error
NaN	NaN	NaN	17	260.0894	Total

The Tukey HSD test was applied to investigate the pairwise differences in more detail. Table 5 shows the Tukey HSD test.

**Table 5:** Tukey HSD pairwise comparison results

P-value	Upper Bound	Lower Bound	Difference	Model 2	Model 1
0.9520	7.5992	0.7667	$-$6.0659	2.0000	1.0000
0.5549	9.5492	2.7167	$-$4.1159	3.0000	1.0000
0.7327	8.7826	1.9500	$-$4.8826	3.0000	2.0000

The results from the ANOVA analysis demonstrate that there is a significant difference in the models and feature extraction methods with regard to classification accuracy. The *P*-value of 0.0200 for the “Columns” (models) indicates that the choice of the model significantly impacts classification performance. Similarly, the *P*-value of 0.0300 for the “Rows” (feature extraction methods) highlights that different feature extraction methods lead to varying classification accuracy, confirming their importance in the process. Besides, the interaction between the models and feature extraction methods shows a significant effect, with a *P*-value of 0.0100. This suggests that the combined use of specific models and feature extraction techniques has a notable influence on classification accuracy. These findings demonstrate that both the model selection and feature extraction method, as well as their interaction, play key roles in determining classification performance.

## Findings and Discussions

The proposed deep learning neural network method, integrated with PSO and ACO, demonstrates significant potential for diagnosing leukemia cell disease from microscope images. A comparative analysis of PSO and ACO methods with machine learning classifiers (SVM, K-NN, and DT) and deep learning architectures (GoogLeNet and ResNet-50) highlights the effectiveness of optimization algorithms in enhancing classification performances.

To assess the differences between groups, a two-way ANOVA test was performed. The results of the ANOVA test showed that at the 0.05 significance level, there were significant differences between the models and feature extraction methods (*P*-value < 0.05). This suggests that a combination of models and feature extraction techniques has a great impact on classification accuracy. Next, to further investigate the differences between pairwise groups, the Tukey HSD test was applied. According to this test, the accuracy of some specific models and feature extraction methods combinations was significantly higher. The results of this study demonstrate that PSO with GoogLeNet achieved the maximum accuracy of 97.4% for SVM, outperforming other classifiers and architectures. At the same time, ACO using ResNet-50 reached a perfect accuracy of 100% for SVM, which suggests potential overfitting. These findings highlight the importance of choosing suitable deep learning architectures and optimization algorithms for feature extraction. Although GoogLeNet was suitable with PSO, ResNet-50 was shown enhanced performance with ACO. This study uses a dataset of 260 images, with 85% for training and 15% for testing. Despite being a standard approach, the limited size of the test set (18 images) raises concerns about generalizability. The need to increase the dataset to improve statistical dependability is indicated by a post hoc power analysis. Standardized Hematoxylin and Eosin (H&E) staining was applied to minimize color and morphological biases, ensuring consistency in image analysis. This preprocessing step proved effective in maintaining segmentation and classification reliability, highlighting the necessity of using similar techniques in future studies. Significant differences in performance are shown by the confusion matrices for the SVM, K-NN, and Decision Tree classifiers. SVM regularly outperformed K-NN and Decision Tree in handling high-dimensional feature spaces. Even though it performs the worst, the Decision Tree remains useful for interpretability-focused applications. PSO and ACO are metaheuristic algorithms that greatly decrease computation times and resource use, making them suitable for large-scale machine learning applications. Furthermore, the incorporation of GWO improved the recognition rate, which indicated that hybrid metaheuristic approaches could yield even better results in future work.

## Limitations and Future Directions

In spite of the encouraging outcomes, some limitations need to be noted. The limited size of the dataset restricts the applicability of our conclusions:

The perfect accuracy achieved by ACO with ResNet-50 highlights potential concerns about overfitting, emphasizing the need for further robustness testing. To improve dependability, future research should expand the dataset for both the application of the k-fold cross-validation technique and its validation against additional external and internal databases, ensuring more accurate results.

Incorporating additional regularization techniques, data enhancement, and early stopping during training could mitigate overfitting risks. Moreover, hybrid metaheuristic strategies that include PSO, ACO, and GWO could offer even more optimization power. Improvement of model interpretability will be another key focus to ensure its use in clinical and diagnostic applications.

By addressing these aspects, valuable insights into leukemia diagnosis using deep learning and metaheuristic optimization techniques are contributed, paving the way for more robust and clinically applicable models.

## Conclusion

In this study, we proposed a deep learning neural network approach that utilizes the particle swarm optimization (PSO) method to diagnose leukemia cell disease from microscope images. The proposed method employs PSO to reduce the feature number and select the most relevant features for optimal performance. Leukemia images are utilized to distinguish between healthy and non-healthy images. Three machine learning algorithms, namely Decision Tree, Support Vector Machine, and K-NN methods are employed to evaluate the performance of the proposed approach.

The experimental results indicate that the ACO feature extraction method outperforms the PSO method in accuracy, with SVM achieving a high score of 100% with ResNet-50. However, overfitting may be a concern. Comparing GoogLeNet and ResNet with PSO and ACO, GoogLeNet performs better with PSO while ResNet performs better with ACO. As seen from the tables, the PSO method yields higher performance than the ACO method, and using GoogLeNet with PSO has yielded better outcomes. The proposed approach achieved accuracies of 97.4%, 92.3%, and 85.9% for SVM, K-NN, and Decision Tree methods, respectively, with GoogLeNet as revealed by the experimental results.

## Data Availability

Dataset is publicly available [24].
